# CHRONIC CONDITION DISCORDANCE WITHIN OLDER COUPLES: IMPLICATIONS FOR HEALTH AND WELL-BEING

**DOI:** 10.1093/geroni/igad104.1594

**Published:** 2023-12-21

**Authors:** Courtney Polenick, Kira Birditt

**Affiliations:** University of Michigan, Ann Arbor, Michigan, United States; Institute for Social Research, University of Michigan, Ann Arbor, Michigan, United States

## Abstract

Nearly half of U.S. adults aged 45-64 and over 80% of those 65 and older have two or more potentially debilitating chronic conditions such as arthritis, diabetes, and heart disease. Chronic condition discordance (i.e., the extent that two or more conditions have non-overlapping self-management requirements) increases care complexity and can make multiple chronic conditions more difficult to manage. Most research on multimorbidity and chronic condition discordance focuses on individuals, however older adults with chronic conditions often have a spouse or partner with chronic health problems that further complicate self-care. Our dyadic concordance model of multimorbidity posits that chronic condition discordance at the individual level (i.e., discordance in two or more chronic conditions within individuals) and the couple level (i.e., discordance in two or more chronic conditions between spouses) has adverse implications for the psychological well-being and self-management behaviors of both partners. We discuss key findings that support this model using longitudinal data from the Health and Retirement Study, which include the implications of individual-level and couple-level chronic condition discordance for depressive symptoms, physical activity, alcohol use, and global and health-related perceived control over time. We then describe how these findings inform clinical care and interventions to improve the health and well-being of couples living with multiple chronic conditions. Finally, we propose future directions to build on and expand the dyadic concordance model of multimorbidity and its use in understanding how older adults manage multiple chronic conditions within the context of their close relationships.

